# Ischaemic heart disease during pregnancy or post-partum: systematic review and case series

**DOI:** 10.1007/s12471-015-0677-6

**Published:** 2015-04-14

**Authors:** H. Lameijer, M.A.M. Kampman, M.A. Oudijk, P.G. Pieper

**Affiliations:** 1 Department of Cardiology, University Medical Centre Groningen, University of Groningen, Hanzeplein 1, 9700 RB Groningen, The Netherlands; 2 Department of Emergency Medicine, University Medical Centre Groningen, University of Groningen, Groningen, The Netherlands; 3 The Netherlands Heart Institute (ICIN), Utrecht, The Netherlands; 4 Department of Obstetrics, University Medical Centre Utrecht, University of Utrecht, Utrecht, The Netherlands

**Keywords:** Ischemic heart disease, Pregnancy, Maternal

## Abstract

The risk of manifestations of ischaemic heart disease (IHD) in fertile women is elevated during pregnancy and the post-partum period. With increasing maternal age and a higher prevalence of cardiac risk factors, the incidence of IHD during pregnancy is rising. However, information in the literature is scarce. We therefore performed a retrospective cohort study and systematically reviewed the overall (1975–2013) and contemporary (2005–2013) literature concerning IHD presenting during pregnancy or in the post-partum period. We report two cases of IHD with atypical presentation during pregnancy or post-partum. In our review, we describe 146 pregnancies, including 57 contemporary cases (2005–2013). Risk factors for IHD were present in 80 %. Of the cases of IHD, 71 % manifested in the third trimester or the post-partum period, and 95 % presented with chest pain. The main cause was coronary dissection (35 %), or thrombus/emboli (35 %) in the more contemporary group. Maternal mortality was 8 % (6 % in the contemporary group), and the main cardiac complication was ventricular tachycardia (*n* = 17). Premature delivery rate was 56 %, and caesarean section was performed in 57 %. Perinatal mortality was 4 %. In conclusion, IHD during pregnancy or in the post-partum period has high maternal mortality and morbidity rates. Also, premature delivery and perinatal mortality rates are high.

**Electronic supplementary material:**

The online version of this chapter (doi:10.1007/s12471-015-0677-6) contains supplementary material, which is available to authorized users.

## Introduction

Cardiovascular disease (CVD) is the leading cause of death in men and women in the Western world [1–3]; 50 % is due to ischaemic heart disease (IHD) [1]. Although pre-menopausal women are relatively protected against atherosclerosis by their hormonal status, the risk of manifestations of IHD is increased during pregnancy and in the post-partum period [4–6]. This is due to cardiovascular and haemodynamic changes and hypercoagulability occurring during pregnancy [7–9]. CVD is the leading cause of indirect maternal death during pregnancy in Western countries with IHD, including acute myocardial infarction (AMI) as a frequent underlying disease [10, 11]. Previous studies estimated an incidence of IHD during pregnancy of 2.8–6.2 per 100,000 deliveries, 3–4 times higher than the incidence found in non-pregnant women of reproductive age [4, 12].

Increasing maternal age and deteriorating lifestyle choices lead to a higher incidence of cardiac risk factors. Consequently, the incidence of IHD during pregnancy will increase worldwide [13, 14]. However, information about IHD presenting during pregnancy is scarce. Incomplete information is available concerning aetiology of IHD, time of presentation and maternal and offspring outcomes [4–6]. We therefore present two cases of women in whom IHD presented during pregnancy or the post-partum period. Furthermore, we systematically reviewed the literature about IHD presenting during pregnancy or in the post-partum period. Additionally, we will present a significant subset of contemporary cases separately.

## Methods

For our case series, we performed a retrospective cohort study. All data were obtained by a systematic search of databases and matching of cardiology department and gynaecology department databases in the University Medical Centre Groningen, Amsterdam Medical Centre and University Medical Centre Utrecht, all in the Netherlands. Diagnostic database matching codes were angina pectoris, ST-segment elevation myocardial infarction (STEMI), non-STEMI, follow-up after myocardial infarction, follow-up after coronary artery bypass grafting and follow-up after percutaneous intervention (PCI). Women who presented with a first manifestation of IHD after conception until 6 weeks post-partum in a 10-year period (2002–2012) were included, regardless of duration, outcome and course of the pregnancy. IHD was defined according to European Society of Cardiology (ESC)/American College of CArdiology/American Heart Association criteria [15]. Women with significant congenital coronary abnormalities were excluded. Retrospective cohort studies do not need to be approved by the institutional review board in the Netherlands.

For our systematic review, we used the Preferred Reporting Items for Systematic Reviews and Meta-Analyses statement protocol [16]. We researched the MEDLINE Public Database for all studies dated until 10 April 2013. Search terminology was Myocardial ischemia and Pregnancy, both in MeSH terms (‘Myocardial Ischemia’ [MeSH] AND ‘Pregnancy’ [ MeSH]) and full text (Myocardial ischemia AND pregnancy). The filters Humans, Case Reports, Meta-Analysis, Clinical Trial, Randomized Controlled Trial, Dutch, English, German, Female, MEDLINE, Adult: 19+ years and Adolescent: 13–18 years were activated. We only included studies written in English, German and Dutch to reduce misinterpretation of data. Systematic reviews were excluded, but new cases described in reviews were included. Cases described before 1975 were excluded. We included all online available articles, either from open-access publishing or availability provided by the University Medical Centre Groningen. Articles describing myocardial ischaemia before pregnancy, ischaemia induced by medication or pheochromocytoma or that caused by Kawasaki’s or Takotsubo syndrome were excluded.

In both our case series as well as our systematic review, we collected data concerning the timing, cause and treatment of IHD, comorbidities, risk factors for IHD and maternal cardiac and obstetric outcome as well as offspring outcome. Prematurity of the foetus was defined as birth at < 37 weeks of gestation, low birth weight was defined as weight < 2500 g and small for gestational age was defined as birth weight < 10th percentile. Perinatal mortality was defined as offspring death from 20 weeks of gestation up to 7 days post-partum. We described cases published in or after 2005 and not included in the latest review [6] separately, and we compared these contemporary cases with previous literature. Statistical analysis was performed using IBM SPSS Statistics Premium V 20 for Windows (IBM Corp., Released 2011; IBM SPSS Statistics for Windows, version 20.0; Armonk, NY, USA). Missing data were excluded for analysis. Continuous data are presented as means with standard deviation (SD) or median with interquartile range (IQR) depending on their distribution. Absolute numbers and percentages were presented for categorical data. For comparison of categorical variables, the Fisher’s exact test or chi-square test was used.

## Results

### Case series

We identified two cases matching our inclusion and exclusion criteria.

Our first case is a 25-year-old woman of Hispanic descent, with one previous miscarriage (gravida (G)2, para (P)0). The patient was severely obese with a body mass index of 39 kg/m^2^. She had a history of a transient ischaemic attack, suspected antiphospholipid syndrome and mitral valvuloplasty for mitral regurgitation due to non-bacterial endocarditis. She was referred to the cardiologist for pre-pregnancy counselling. When she was pregnant, her vitamin K antagonist was replaced by acetylsalicylic acid and a full dose of low-molecular-weight heparin during pregnancy until the fifth day post-partum. At 27 weeks of gestation, she presented with complaints of upper abdominal pain. She was diagnosed with pre-eclampsia complicated by haemolysis, elevated liver enzymes and low platelet (HELLP) syndrome (alanine aminotransferase: 143 U/l, thrombocytes: 128 × 10^9^/l). Foetal ultrasonography showed normal growth and the foetal condition judged by cardiotocography was well. The patient was treated with labetalol and magnesium sulphate (MgSO_4_). At 29 + 3 weeks of gestation, her condition worsened, and a caesarean section was performed. She delivered a baby girl of 1067 g (50th percentile) with an Apgar score of 6 at 5 min. The neonate had to be admitted to the neonatal intensive care unit because of prematurity. Three days post-partum, the mother presented with syncope. Chest pain was not reported. Electrocardiographic (ECG) monitoring showed ST-segment depression and Q waves, suggesting inferolateral AMI, which was confirmed by elevated troponin T (5.96 μg/l; normal: < 0.014 μg/l). Her coronary angiogram showed no abnormalities. The AMI was presumably caused by a thrombus, embolism or coronary spasm. Both mother and neonate survived. Her medication was upgraded to a beta-blocker, angiotensine I converting enzyme inhibitor, statin, acetylsalicylic acid and vitamin K antagonist. Echocardiography at 6 months showed a mildly reduced left ventricular function. The diagnosis of antiphospholipid syndrome was confirmed.

Our second case is a 42-year-old woman, G1P0. She had a history of insulin-dependent diabetes mellitus, pulmonary embolism and a positive family history for IHD. She was referred to a university hospital by an obstetric clinic at 15 weeks of gestation because of an episode of ventricular tachycardia. Her ECG suggested anterior AMI, which was confirmed by raised troponin (37.77 μg/l) and creatine kinase (2239 U/l) levels. Her coronary angiogram revealed atherosclerotic occlusion of the left main coronary artery. She was treated with stenting of the left coronary artery and medically with acetylsalicylic acid, beta-blocker, clopidogrel and subcutaneous heparin. At 37 weeks of gestation, intrauterine growth retardation and placental insufficiency were suspected. The decision was made to perform an elective caesarean section. She delivered a live born neonate at 37 + 5 weeks. Neonatal Apgar score at 5 min was 10, and birth weight was 2405 g, which is at the fifth percentile for gestational age. Histological examination of the placenta showed a small placenta (weight: < 10th percentile), with diffuse ischaemia, consistent with placental insufficiency. A statin was added to the maternal medical regimen during the post-partum period. Maternal ventricular function remained normal during 6 months of follow-up. A stress test and nuclear scan revealed no signs of recurrent ischaemia. The neonate did well.

### Systematic review

**Fig. 1:**
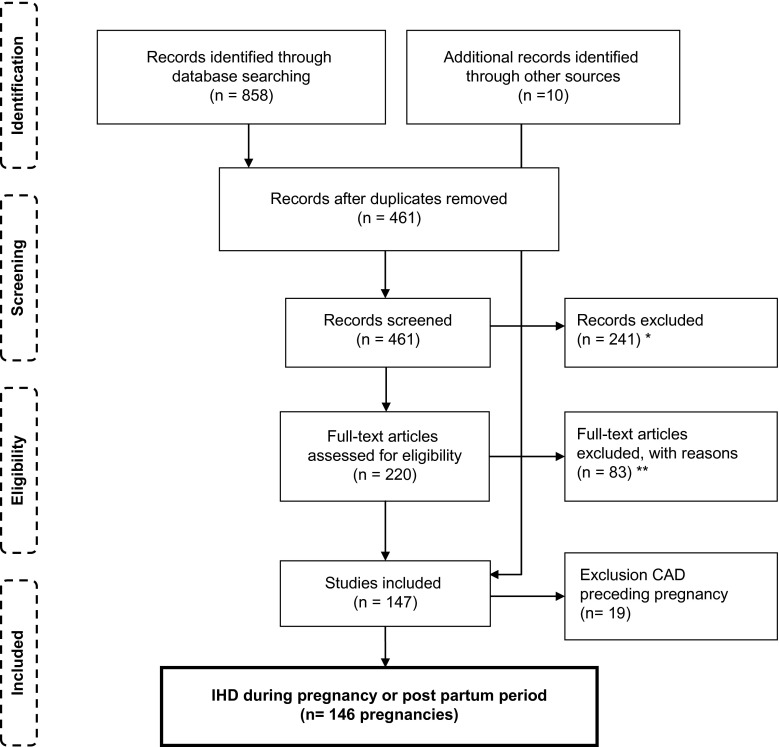
Flow diagram for inclusion of literature (*IHD* ischaemic heart disease, *CAD* coronary artery disease, *exclusion based on abstract and title, **non-available articles were excluded)

We found 128 articles describing IHD presenting during pregnancy and in the post-partum period, with a total of 146 pregnancies, including 6 twin pregnancies and 1 triplet pregnancy. Inclusion is schematically presented in Fig. 1. We excluded several studies for statistical analysis because of incomplete individual data concerning both cardiac and obstetric outcomes. The results of these studies are summarised and compared with our results in a table and are discussed in our discussion section [4–6, 17–19]. All articles included were published between 1978 and 2012 and are presented in supplemental Table S1, which is available online.

#### Baseline characteristics

Baseline maternal characteristics are presented in Table 1.

**Table 1:** Baseline characteristics of women with ischaemic heart disease presenting during pregnancy, according to the literature included in our review

	*N*	*Mean (SD)*
Pregnancies	146^a^	
Age of woman	145	33.2 (5.8) years
Gravida	105	3.1 (2.0)
Parity	97	1.8 (1.6)
*Coronary risk factors*	*N (women)*	*Percentage*
Smoking	50	40
Dyslipidaemia	26	21
Pre-pregnancy hypertension	24	20
Family history	22	18
Obesity (pre-pregnancy body mass index > 30 kgm^2^)	17	15
Diabetes mellitus	9	8
Use of illegal drugs before event (cocaine)	3	3
One or more risk factors	80	63
Two or more risk factors	44	34
*Cardiac history*		
Chest pain	11	9
Valvular lesions	6	5
Heart failure	2	2
Supra-ventricular tachycardia	2	2
Atrial fibrillation	2	2
Pulmonary embolus	1	1
*Concurrent conditions*		
Thyroid disease	4	3
Factor V Leiden	4	3
Thrombophillia	2	2
Connective tissue disease	2	2
Infectious disease	1	1
Other	19	12

Missing data were excluded for analysis

*SD* standard deviation

^a^Including six twin pregnancies and one triplet pregnancy

#### IHD, characteristics and treatment

Characteristics of IHD during pregnancy, delivery or in the post-partum period are reported in Table 2. Comparison with other studies and characteristics of the contemporary group can be found in Table 3. All women experienced symptoms suggestive of AMI. In 89 % of cases, ST-segment deviation was seen on the ECG. In contrast to the overall group of women with IHD during pregnancy, where dissection was the most prevalent cause of IHD, in the contemporary group (*n* = 57), the incidences of thrombus or embolism and of dissection were comparable (20 versus 18 women; Table 2). Of the women who had AMI due to atherosclerosis, 93 % had one or more risk factors for IHD, compared with 43 % of the women who had AMI caused by coronary dissection (*p* < 0.001) and 68 % of women with thrombus or emboli (*p* < 0.01).

**Table 2:** Details of ischaemic heart disease and offspring outcomes in 146 pregnancies (including 6 twin pregnancies and one triplet pregnancy) according to the literature included in our review

Presenting symptoms	Number of pregnancies	Percentage
Chest pain	131	95
Dyspnoea	38	36
Syncope	10	9
Dizziness	10	10
Heart failure syndrome	6	6
Palpitations	1	1
Exercise intolerance	4	4
No symptoms	0	0
*Location of AMI*		
Anterior, anteroseptal or anterolateral	80	67
Inferior, inferoposterior or inferolateral	22	19
Other	13	14
*Presumed aetiology of IHD*		
Coronary dissection	46	35
Thrombus/embolism	33	25
Atherosclerosis/stenosis	31	24
Coronary spasm/other/unexplained	20	15
*Timing of AMI*		
During pregnancy, first trimester	9	6
During pregnancy, second trimester	22	15
During pregnancy, third trimester	56	38
During post-partum period	50	33
During delivery	7	5
During pregnancy, unknown	2	1
*Offspring outcome*		
Live born	128	96
Perinatal death	5	4
Premature birth	55	56
Low birth weight	19	40
Small for gestational age	1	2
*Apgar score (at 5 min)*		
< 7	5	16
7–10	27	84

Perinatal death stands for intrauterine foetal death and stillborn

Premature birth is defined as birth at < 37 weeks of gestation, low birth weight is defined as weight <2500 g, small for gestational age is defined as birth weight < 10th percentile

Missing data were excluded for analysis

*AMI* acute myocardial infarction, *IHD* ischaemic heart disease

**Table 3:** Overview and comparison of data described in the main literature concerning ischaemic heart disease during pregnancy and this study

Literature (women)	Ladner et al. [5] (*N* = 151)	Satoh et al. [17] (*N* = 62)	James et al. [4] (*N* = 859)	Roth et al. [6] (*N* = 103)	This study *Overall* (*N* = 146)	This study *Contemporary only* (*N* = 57)
Years of inclusion	1991–2000	1981–2001	2000–2002	1995–2005	1978–2012	2005–2012
Mean age of women (years)	31–35	33	33	33	33.2	33.5
Most common timing of coronary event (*N*)	Post-partum (62)	Post-partum (28)	During pregnancy, *not specified* (626)	During pregnancy, *not specified* (46)	During pregnancy, third trimester (56)	During pregnancy, third trimester (25)
Most common location of AMI (*N*)	^a^	Including anterior wall (31)	Including anterior wall (215)	Including anterior wall (73)	Including anterior wall (80)	Including anterior wall (26)
Most common aetiology of IHD (*N*)	^a^	Coronary dissection (14)	^a^	Coronary stenosis (41)	Coronary dissection (46)	Thrombus/embolism (20)
Most common risk factor for IHD (*N*)	HT^a^	Smoking (9)	^a^	Smoking (46)	Smoking (40)	Smoking (17)
Maternal mortality (*N*)	7.3 % (11)	3.2 %^a^ (2)	5.1 % (44)	11 % (11)	8 % (11)	6 % (3)
Most common (other) maternal cardiac complication (*N*)	^a^	Cardiogenic shock (5), VF/VT (5), HF (5)	^a^	HF (9)	VT (17)	VT (3)
Most common maternal obstetric complication (*N*)	PIH (24)	PPH (1)	PPH^a^	Pre-eclampsia (6)	PIH (46)	PIH (6)
Caesarean section rate (*N*)	^a^	^b^	^a^	38 % (39)	57 % (75)	67 % (36)
Perinatal mortality (*N*)	^a^	^a^	^a^	9 % (6)	4 % (5)	6 % (3)
Most common offspring complication (*N*)	Prematurity^a^	Threatened premature delivery (3)	^a^	^a^	Prematurity (55)	Prematurity (28)

Offspring mortality is defined as offspring death from 20 weeks of gestation up to 7 days post-partum; prematurity is defined as birth at < 37 weeks of gestation

Missing data were excluded for analysis

^a^Unknown or not clearly reported data

^b^At least seven women; incompletely documented

*AMI* acute myocardial infarction; *HF* heart failure; *HT* essential hypertension; *IHD* ischaemic heart disease; *PIH* pregnancy-induced hypertensive disorders, including pre-eclampsia, eclampsia and haemolysis, elevated liver enzymes and low platelets syndrome; *PPH* post-partum haemorrhage; *VF* ventricular fibrillation; *VT* ventricular tachycardia

**Fig. 2:**
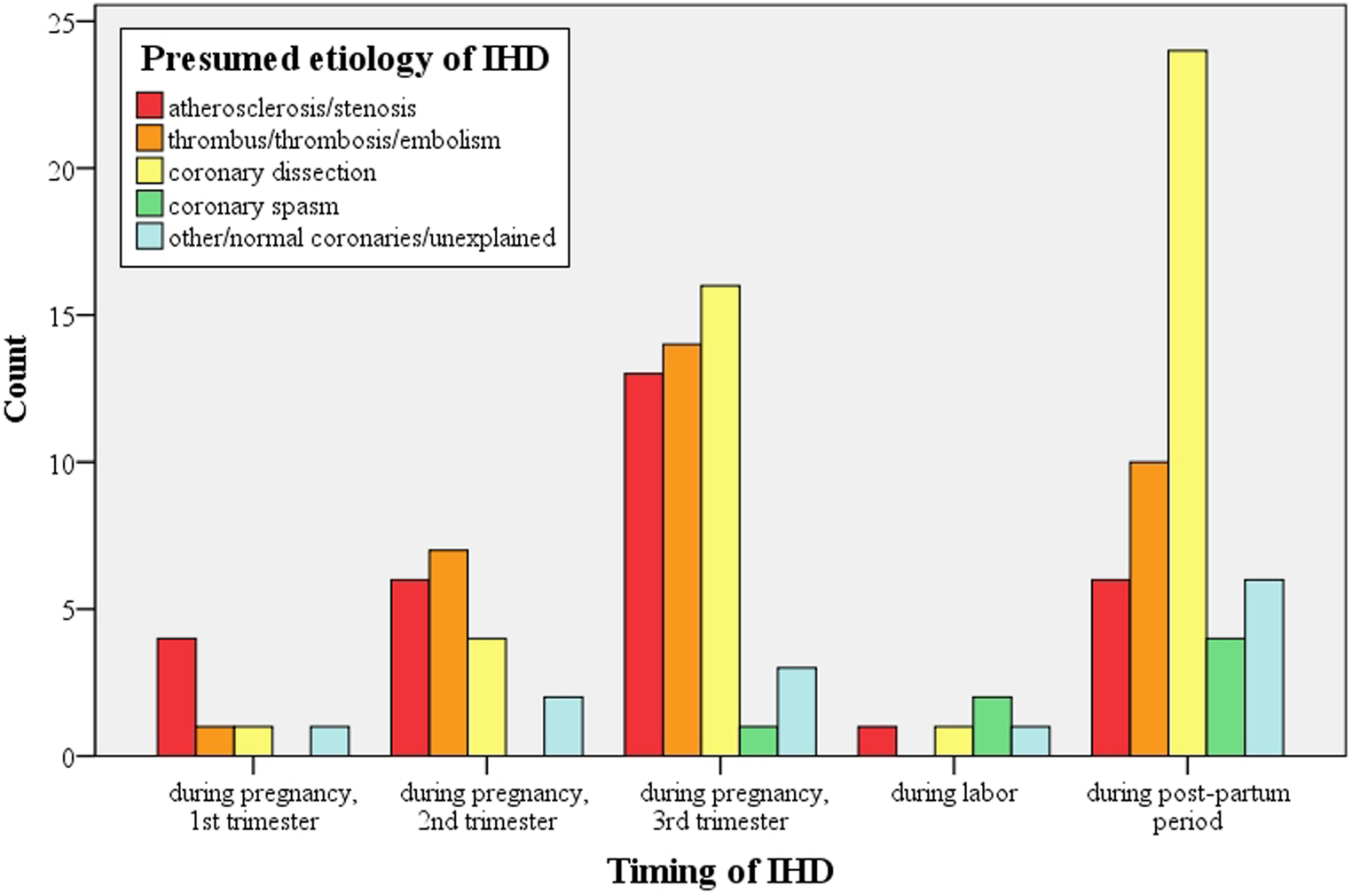
Aetiology of ischaemic heart disease depending on the time of presentation during pregnancy (*IHD* ischaemic heart disease)

The aetiology differed depending on the time of presentation during pregnancy (Fig. 2). In all, 87 % of the cases of coronary dissection presented in the third trimester or post-partum period. Atherosclerosis peaked in the third trimester (42 % of all cases of atherosclerosis), whereas AMI with normal coronaries or caused by thrombosis or emboli was independent of the stage of pregnancy. Most women were treated non-invasively (*n* = 50) or with PCI (*n* = 47). Coronary artery bypass surgery was performed in 22 women; in 34 women, therapy was not clearly reported.

#### Maternal outcome

Comparison with other studies and characteristics of the contemporary group can be found in Table 2.

##### Cardiac outcome

Seventeen women had had an episode of ventricular tachycardia, mostly as a presenting symptom. Additionally, six women suffered an episode of cardiac arrest. In six women, IHD was complicated by heart failure; cardiogenic shock occurred in one woman. Ten women had to be intubated during hospitalisation, of whom four did not survive. In total, 11 deaths were reported (8 %). We found 6 % mortality in the contemporary group, compared with 9 % in the group published before 2005 (*p* = 0.337).

##### Obstetric outcome

Hypertensive disorders during pregnancy were reported in 28 women (18 %), progressing to pre-eclampsia/eclampsia in 15 women (10 %) and HELLP syndrome in 3 (2 %) women. These pregnancy-related hypertensive disorders were not found more frequently in women with coronary artery dissection. Delivery was mainly by caesarean section (57 %). The caesarean section rate was not significantly different in women who presented with AMI during pregnancy (62 %) compared with women who had their AMI in the post-partum period (44 %, *p* = 0.08). In four women post-partum haemorrhage was described.

##### Late complications

In 49 % of the women, 6-month follow-up was reported. Of these women, 64 % had no complications during follow-up; in 21 %, a reduced cardiac function was reported. One woman needed a heart transplantation for progressive cardiac dysfunction. A few reported recurrent angina (*n* = 5) or coronary pseudoaneurysm/aneurysm (*n* = 2).

#### Offspring outcome

Offspring outcome is summarised in Table 3. Perinatal mortality was 4 %. Reported causes of mortality included maternal mortality (*n* = 2), non-cardiac congenital malformations, prematurity and suspected reduced placental perfusion during cardiopulmonary bypass surgery. Overall median time of delivery was 36 (IQR: 34–38) weeks. In all, 56 % of the neonates were delivered prematurely (*n* = 55), which was significantly related to a higher rate of caesarean section (*p* = 0.012). Prematurity rate was 54 % in IHD manifesting during pregnancy and 60 % in IHD manifesting during delivery or in the post-partum period. Mean neonatal birth weight was 2645 g (SD: 932 g), and around the 50th percentile for gestational age in almost all neonates. Low birth weight was reported in 19 patients (missing data in *n* = 107). Only one neonate was small for gestational age. Nine neonates were reported to be admitted to the neonatal intensive care unit. The main reason was prematurity.

## Discussion

In this case series and review, we add a significant number of new cases compared with previous reviews [6, 17], including 57 contemporary cases published after 2004. Our review also adds more detailed data concerning aetiology of IHD and maternal and offspring outcome. Our review confirmed that IHD is rare in pregnancy. Pregnant women with IHD present mostly with chest pain (95 %) in the third trimester or post-partum period. Risk factors are invariably present in atherosclerotic disease but less often in thrombotic disease and coronary dissection. Maternal and foetal complication rates, including maternal mortality, are high.

Though IHD is the most common cause of maternal cardiac death in the UK,the estimated incidence of non-fatal IHD in the UK is only 0.7 per 100,000 maternities [11, 20]. As we only found two cases of IHD during pregnancy in our systematic search of three large university hospital databases, IHD presenting during pregnancy is also rare in the Netherlands. This is in line with a recent prospective Dutch study that reported an incidence of 0.005 % [18].In a worldwide registry describing 1321 pregnancies in women with heart disease, only 4 women with a first manifestation of IHD were reported [19].

### Risk factors

Risk factors for IHD were present in both women in our case series and in the majority of the women in our review. This is in line with previous literature [4–6, 17–19]. Also, this indicates a large impact of lifestyle factors on IHD during pregnancy, as also described in the UK maternal death report [11]. In line with a recently published study in Japanese women, we observed less risk factors in women with coronary artery dissection or thrombus/emboli than in women with atherosclerosis as a cause of AMI, suggesting a different pathophysiology [17].

### IHD, characteristics and treatment

Women in our review had a relatively high age compared with the average age at time of pregnancy in the USA [21]. This is comparable with previous literature [5, 6, 19]. Coronary dissection is rare outside pregnancy, but it was the main cause of IHD in the women in our review. However, in our more recent cases, thromboembolic coronary events were seen equally frequently. Thromboembolic events may be largely attributed to pregnancy and its hypercoagulable state. Relatively high rates of coronary dissection during pregnancy have previously been described [17]. In line with previous studies, most cases of AMI presented in the third trimester and post-partum period [4, 5, 17]. Especially coronary dissection peaked in these periods, which may be explained by progressive connective tissue weakening and therefore susceptibility for dissection in late pregnancy. Pregnancy-related hypertensive disorders did not seem to contribute to the high incidence of coronary dissection, nor did inherited connective tissue diseases.

In contrast to the atypical presentation of our two cases, in our review, chest pain was the main presenting symptom of IHD. Most of the cases of AMI in our review could be detected on the ECG. In the UK maternal death report, substandard quality of care was observed in 46 % of the women who died due to IHD. This often included delay of cardiac evaluation because IHD was not considered to be a possible diagnosis. Delayed recognition of IHD during pregnancy was also described in a recent Dutch study [18]. In pregnant women with chest pain, especially when they have risk factors, IHD should be considered and an ECG and laboratory investigation should be performed.

### Maternal outcome

A relatively large percentage of women in our systematic review presented with serious complications directly due to AMI, including heart failure, a complication frequently seen in pregnant women with cardiac disease [19, 22]. In line with previous literature, mortality rate during pregnancy in women with IHD was higher than in pregnant women with cardiac disease overall [5, 6, 17, 19]. The slightly lower mortality rate in contemporary cases may be explained by improvement of coronary care.

Pregnancy-related hypertensive disorders found to be associated with IHD during pregnancy were seen more frequently compared with pregnant women with non-ischaemic heart disease [19, 23–25]. We observed a very high caesarean section rate of 57 %, which was even higher in contemporary cases. This is higher than the caesarean section rate in healthy pregnant women (21 %) [26], higher than in a previous review [6] and higher than in women with congenital or valvular heart disease (38 and 42 %). However, caesarean section rate was comparable with women with cardiomyopathy (58 %) or known IHD (60 %) [19]. The high caesarean section rate was related to the high rate of premature deliveries. This high premature delivery rate and high premature caesarean section rate may be due to several factors, such as the high rate of hypertensive disorders or maternal cardiac reasons for early pregnancy termination. Also, they may possibly be related to physicians’ reluctance for vaginal delivery in women with a recent myocardial infarction. Post-partum haemorrhage was described in 3 % of the women. This is comparable with women with known cardiac disease [23, 27–29] and only slightly more than in the general population [30, 31].

### Offspring outcome

Perinatal mortality was increased at 4 % and mainly attributable to maternal death and prematurity. Prematurity rate was 3.4–13.2 times higher compared with the prematurity rates in healthy pregnant women [32–35]. Furthermore, it was even high compared with women with non-ischaemic heart disease [19, 23, 36].A high rate of induced early deliveries may be part of the explanation. However, prematurity rate was comparable in IHD manifesting during pregnancy with IHD manifesting during labour or in the post-partum period, suggesting an additional mechanism for the high prematurity rates. Interestingly, in contrast to reports in women with non-ischaemic heart disease, the incidence of small for gestational age was not elevated [25, 36, 37].

## Limitations

By only including online available articles and articles in English, Dutch or German, we may have missed data. In our review, analysis was performed by excluding missing data, which might have led to deformation of the results. This is particularly important when missing data were abundant (i.e. cardiac function during follow-up). Also, publication bias and selective reporting within studies, which could affect the cumulative evidence, could not be minimised. Because follow-up was insufficiently reported and limited, (late) maternal complications, including death, may have been underestimated.

## Conclusions

In contrast to the atypical presentation in our case series, IHD during pregnancy mainly presents with chest pain and during the third trimester or the post-partum period. The main causes are coronary dissection and, in more recent cases, thrombus and embolism. Risk factors for IHD were present in most women with atherosclerotic disease, but less often in women with coronary dissection or thrombosis/embolism. IHD during pregnancy or in the post-partum period has a high maternal mortality rate and high maternal cardiac complication rates. Perinatal mortality and premature birth are increased in women with IHD and related to high caesarean section rate. Clinicians should seriously consider IHD when a pregnant woman presents with chest pain, in particular, in women with known risk factors for IHD. However, atypical presentation (i.e. collapse) is also possible.

### Funding

None.

## Electronic supplementary material

(PDF 108 KB)
