# Supplementary material for: Ischaemic heart disease during pregnancy or post-partum: systematic review and case series
Source: Neth Heart J. 2015 Apr 14;23(5):249–57. doi: 10.1007/s12471-015-0677-6 (PMC4409591; doi:10.1007/s12471-015-0677-6)
Supplement: Supplementary file 1 — (PDF 108 KB) [file 12471_2015_677_MOESM1_ESM.pdf]

Supplemental Table S1. Characteristics of included literature.

**Authors**	**Year**	**Type of study**
Aalders K., A. et al.[1]	1998	case series
Agostoni P. et al.[2]	2004	case report
Aliyary,S. et al.[3]	2007	case report
Allen,J.N. et al.[4]	1990	case report
Arimura T. et al.[5]	2009	case report
Ascarelli,M.H. et al.[6]	1996	case report
Babic,Z. et al.[7]	2011	case report
Badui,E. et al.[8]	1994	case series
Balmain,S. et al.[9]	1997	case report
Baskurt,M. et al.[10]	2012	case report
Bauer,M.E. et al.[11]	2012	case report
Beary,J.F. et al.[12]	1979	case series and review
Bornstein,A. et al.[13]	1984	case report
Boyer,W.B. et al.[14]	2011	case report
Boztosun,B. et al.[15]	2008	case report
Brahim,Y.B. et al.[16]	2008	case report
Brandenburg,V.M. et al.[17]	2004	case report
Bucciarelli,E. et al.[18]	1998	case report
Chabrot,P. et al.[19]	2009	case report
Chant,G.N.[20]	1979	case series and review
Chen,Y.C. et al.[21]	2009	case report
Cohen,W.R. et al.[22]	1983	case report

Collins,J.S. et al.[23]	2002	case report and review
Collyer,M. et al.[24]	2004	case report
Cowan,N.C. et al.[25]	1988	case report
Craig,S. et al.[26]	1999	case report
Cuthill,J.A. et al.[27]	2005	case report and review
Dhawan,R. et al.[28]	2011	case report
Dhawan,R. et al.[29]	2002	case report
Diessner,J. et al.[30]	2011	case report
Dwyer,B.K. et al.[31]	2005	case report
Ehya,H. et al.[32]	1980	case report
Eickman,F.M.[33]	1996	case report
Elming,H. et al.[34]	1999	case report and review
Emori,T. et al.[35]	1993	case report
Eom,M. et al.[36]	2005	case report
Eriksson,U. et al.[37]	1999	case report
Esinler,I. et al.[38]	2003	case report
Fayomi,O. et al.[39]	2007	case report
Frey,B.W. et al.[40]	2006	case report
Garry,D. et al.[41]	1996	case report
Garvey,P. et al.[42]	1998	case report
Ginwalla,M. et al.[43]	2010	case report
Giudici,M.C. et al.[44]	1989	case report
Hamada,S. et al.[45]	1996	case report
Hameed,A.B. et al.[46]	2000	case series

Hands,M.E. et al.[47]	1990	case series
Hankins,G.D. et al.[48]	1985	case series and review
Hoppe,U.C. et al.[49]	1998	case report
Houck,P.D. et al.[50]	2012	case report
Iaccarino,D. et al.[51]	2010	case report
Iadanza,A. et al.[52]	2007	case report and review
Janion,M. et al.[53]	2007	case series
Jimenez Valero,S. et al.[54]	2005	case series
Jungbluth,A. et al.[55]	1988	case report
Kamran,M. et al.[56]	2004	case report
Kearney,P. et al.[57]	1993	case series and review
Klutstein,M.W. et al.[58]	1997	case series
Knoess,M. et al.[59]	2007	case series
Koul,A.K. et al.[60]	2001	case series and review
Kuczkowski,K.M.[61]	2005	case report
Kulka,P.J. et al.[62]	2000	case report
Kurum,T. et al.[63]	2003	case report
Laudanski,K. et al.[64]	2011	case report and review
Lerakis,S. et al.[65]	2001	case report
Liu,S.S. et al.[66]	1992	case report
Livingston,J.C. et al.[67]	2000	case report
Mabie,W.C. et al.[68]	1988	case report
Madu,E.C. et al.[69]	1994	case report and review

Maeder,M. et al.[70]	2005	case report and review
Majdan,J.F. et al.[71]	1983	case report
Mak,K.H. et al.[72]	2004	case report
Makkonen,M. et al.[73]	1995	case report
Marcoff,L. et al.[74]	2010	case report
Martins,R.P. et al.[75]	2010	case report
McAdams,S.A. et al.[76]	1986	case series
McHugh,M.J. et al.[77]	1990	case report and review
McKechnie,R.S. et al.[78]	2001	case report
McKeon,V.A. et al.[79]	1989	case report and review
Moore,A.D. et al.[80]	2012	case report
Movsesian,M.A. et al.[81]	1989	case report
Nabatian,S. et al.[82]	2005	case series
Nallamothu,B.K. et al.[83]	2005	other
Newell,C.P. et al.[84]	2011	case report
O'Donnell,M. et al.[85]	1987	case report
Oki,K.N. et al.[86]	2011	case report
Ottman,E.H. et al.[87]	1993	case report
Pauleta,J.R. et al.[88]	2007	case report
Phillips,L.M. et al.[89]	2006	case report
Pierre-Louis,B. et al.[90]	2008	case report and review
Raber,L. et al.[91]	2011	case report
Rademacher,W. et al.[92]	2010	case report

Rahman,S. et al.[93]	2009	case report
Rajab,T.K. et al.[94]	2010	case report
Ramineni,R. et al.[95]	2010	case report
Rensing,B.J. et al.[96]	1999	case report
Rifai,L. et al.[97]	2011	case report
Sabatine,M.S. et al.[98]	2010	case report
Sage,M.D. et al.[99]	1986	case report
Salam,A.M.[100]	2005	case report
Salem,D.N. et al.[101]	1984	case report
Samuels,L.E. et al.[102]	1998	case report
Saxena,R. et al.[103]	1992	case report
Schiff,J.H. et al.[104]	2007	case report
Schumacher,B. et al.[105]	1997	case report
Sebastian,C. et al.[106]	1998	case report
Shahabi,S. et al.[107]	2008	case series and review
Sharma,A.M. et al.[108]	2011	case report
Shaver,P.J. et al.[109]	1978	case report
Sherif,H.M. et al.[110]	2008	case report
Silberman,S. et al.[111]	1996	case report
Skoura,A. et al.[112]	2008	case report
Spencer,J. et al.[113]	1994	case report
Stefanovic,V. et al.[114]	2004	case report
Tang,A.T. et al.[115]	2004	case report
Tatham,K. et al.[116]	2010	case report

Taylor,G.W. et al.[117]	1993	case report
Togni,M. et al.[118]	1999	case report
Trouton,T.G. et al.[119]	1988	case series
Ulm,M.R. et al.[120]	1996	case report
van de Putte,P. et al.[121]	1995	case series
Varadarajan,P. et al.[122]	2006	case report
Vogiatzis,I. et al.[123]	2010	case report
von Steinburg,S.P. et al.[124]	2011	case report
Webber,M.D. et al.[125]	1997	case report
Wittry,M.D. et al.[126]	1989	case report
Yla-Outinen,A. et al.[127]	1989	case report and review
Zaidi,A.N. et al.[128]	2008	case report

References in table:

1. Aalders K, Huisman A, Bosker HA. Myocardial infarct in the puerperium. Ned Tijdschr Geneeskd. 1998; 142:1103-1105.

2. Agostoni P, Gasparini G, Destro G. Acute myocardial infarction probably caused by paradoxical embolus in a pregnant woman. Heart. 2004; 90:e12.

3. Aliyary S, Mariani MA, Verhorst PM, Hartmann M, Stoel MG, von Birgelen C. Staged therapeutic approach in spontaneous coronary dissection. Ann Thorac Surg. 2007; 83:1879-1881.

4. Allen JN, Wewers MD. Acute myocardial infarction with cardiogenic shock during pregnancy: Treatment with intra-aortic balloon counterpulsation. Crit Care Med. 1990; 18:888-889.

5. Arimura T, Mitsutake R, Miura S, Nishikawa H, Kawamura A, Saku K. Acute myocardial infarction associated with pregnancy successfully treated with percutaneous coronary intervention. Intern Med. 2009; 48:1383-1386.

6. Ascarelli MH, Grider AR, Hsu HW. Acute myocardial infarction during pregnancy managed with immediate percutaneous transluminal coronary angioplasty. Obstet Gynecol. 1996; 88:655-657.

7. Babic Z, Gabric ID, Pintaric H. Successful primary percutaneous coronary intervention in the first trimester of pregnancy. Catheter Cardiovasc Interv. 2011; 77:522-525.

8. Badui E, Rangel A, Enciso R, et al. Acute myocardial infarction during pregnancy and puerperium in athletic women. two case reports. Angiology. 1994; 45:897-902.

9. Balmain S, McCullough CT, Love C, Hughes R, Heidemann B, Bloomfield P. Acute myocardial infarction during pregnancy successfully treated with primary percutaneous coronary intervention. Int J Cardiol. 2007; 116:e85-7.

10. Baskurt M, Ozkan T, Arat Ozkan A, Gurmen T. Acute myocardial infarction in a young pregnant woman. Anadolu Kardiyol Derg. 2010; 10:285-286.

11. Bauer ME, Bauer ST, Rabbani AB, Mhyre JM. Peripartum management of dual antiplatelet therapy and neuraxial labor analgesia after bare metal stent insertion for acute myocardial infarction. Anesth Analg. 2012; 115:613-615.

12. Beary JF, Summer WR, Bulkley BH. Postpartum acute myocardial infarction: A rare occurrence of uncertain etiology. Am J Cardiol. 1979; 43:158-161.

13. Bornstein A, Dalal P, Tischler J, Novack S, Michaelson S. Acute myocardial infarction in a thirty-six year old postpartum female. Angiology. 1984; 35:591-594.

14. Boyer WB, Atalay MK, Sharaf BL. Left main pseudoaneurysm after postpartum coronary dissection. Circ Cardiovasc Interv. 2011; 4:303-305.

15. Boztosun B, Olcay A, Avci A, Kirma C. Treatment of acute myocardial infarction in pregnancy with coronary artery balloon angioplasty and stenting: Use of tirofiban and clopidogrel. Int J Cardiol. 2008; 127:413-416.

16. Brahim YB, Landoulsi H, Yassin A, Falfoul A. Pregnancy complicated by myocardial infarction. Int J Gynaecol Obstet. 2008; 103:65-66.

17. Brandenburg VM, Frank RD, Heintz B, Rath W, Bartz C. HELLP syndrome, multifactorial thrombophilia and postpartum myocardial infarction. J Perinat Med. 2004; 32:181-183.

18. Bucciarelli E, Fratini D, Gilardi G, Affronti G. Spontaneous dissecting aneurysm of coronary artery in a pregnant woman at term. Pathol Res Pract. 1998; 194:137-139.

19. Chabrot P, Motreff P, Boyer L. Postpartum spontaneous coronary artery dissection: A case of pseudoaneurysm evolution detected on MDCT. AJR Am J Roentgenol. 2006; 187:W660.

20. Chant GN. Coronary anatomy in postpartum acute myocardial infarction. Am J Cardiol. 1980; 45:912.

21. Chen YC, Chang YM, Yeh GP, Tsai HD, Hsieh CT. Acute myocardial infarction during pregnancy. Taiwan J Obstet Gynecol. 2009; 48:181-185.

22. Cohen WR, Steinman T, Patsner B, Snyder D, Satwicz P, Monroy P. Acute myocardial infarction in a pregnant woman at term. JAMA. 1983; 250:2179-2181.

23. Collins JS, Bossone E, Eagle KA, Mehta RH. Asymptomatic coronary artery disease in a pregnant patient. A case report and review of literature. Herz. 2002; 27:548-554.

24. Collyer M, Bellenger N, Nachimuthu P, Parasuraman R, Taylor MJ. Postpartum coronary artery dissection. J Obstet Gynaecol. 2008; 28:451-453.

25. Cowan NC, de Belder MA, Rothman MT. Coronary angioplasty in pregnancy. Br Heart J. 1988; 59:588-592.

26. Craig S, Ilton M. Treatment of acute myocardial infarction in pregnancy with coronary artery balloon angioplasty and stenting. Aust N Z J Obstet Gynaecol. 1999; 39:194-196.

27. Cuthill JA, Young S, Greer IA, Oldroyd K. Anaesthetic considerations in a parturient with critical coronary artery disease and a drug-eluting stent presenting for caesarean section. Int J Obstet Anesth. 2005; 14:167-171.

28. Dhawan R, Kacha A, Chaney MA, Fox AA, Wong CA. Case 2--2011: Acute myocardial infarction in a pregnant patient requiring coronary artery bypass graft surgery. J Cardiothorac Vasc Anesth. 2011; 25:353-361.

29. Dhawan R, Singh G, Fesniak H. Spontaneous coronary artery dissection: The clinical spectrum. Angiology. 2002; 53:89-93.

30. Diessner J, Heuer S, Engel JB, et al. Myocardial infarction in the 34th week of gestation: Case report. Z Geburtshilfe Neonatol. 2011; 215:209-211.

31. Dwyer BK, Taylor L, Fuller A, Brummel C, Lyell DJ. Percutaneous transluminal coronary angioplasty and stent placement in pregnancy. Obstet Gynecol. 2005; 106:1162-1164.

32. Ehya H, Weitzner S. Postpartum dissecting aneurysm of coronary arteries in a patient with sarcoidosis. South Med J. 1980; 73:87-88.

33. Eickman FM. Acute coronary artery angioplasty during pregnancy. Cathet Cardiovasc Diagn. 1996; 38:369-372.

34. Elming H, Kober L. Spontaneous coronary artery dissection. case report and literature review. Scand Cardiovasc J. 1999; 33:175-179.

35. Emori T, Goto Y, Maeda T, Chiba Y, Haze K. Multiple coronary artery dissections diagnosed in vivo in a pregnant woman. Chest. 1993; 104:289-290.

36. Eom M, Lee JH, Chung JH, Lee H. An autopsy case of postpartum acute myocardial infarction associated with postpartum ergot alkaloids administration in old-aged pregnant women. Yonsei Med J. 2005; 46:866-869.

37. Eriksson U, Atar D, Krahenmann F, et al. Profound ECG abnormalities during emergency cesarean section in a patient with pre-eclampsia. Scand Cardiovasc J. 1999; 33:183-186.

38. Esinler I, Yigit N, Ayhan A, Kes S, Aytemir K, Acil T. Coronary artery dissection during pregnancy. Acta Obstet Gynecol Scand. 2003; 82:194-196.

39. Fayomi O, Nazar R. Acute myocardial infarction in pregnancy: A case report and subject review. Emerg Med J. 2007; 24:800-801.

40. Frey BW, Grant RJ. Pregnancy-associated coronary artery dissection: A case report. J Emerg Med. 2006; 30:307-310.

41. Garry D, Leikin E, Fleisher AG, Tejani N. Acute myocardial infarction in pregnancy with subsequent medical and surgical management. Obstet Gynecol. 1996; 87:802-804.

42. Garvey P, Elovitz M, Landsberger EJ. Aortic dissection and myocardial infarction in a pregnant patient with turner syndrome. Obstet Gynecol. 1998; 91:864.

43. Ginwalla M, Pillai D, Gandhi S. Use of intra-aortic balloon counterpulsation during emergent cesarean section in a pregnant patient with myocardial infarction. J Invasive Cardiol. 2010; 22:E104-6.

44. Giudici MC, Artis AK, Webel RR, Alpert MA. Postpartum myocardial infarction treated with percutaneous transluminal coronary angioplasty. Am Heart J. 1989; 118:614-616.

45. Hamada S, Hinokio K, Naka O, Higuchi K, Takahashi H, Sumitani H. Myocardial infarction as a complication of pheochromocytoma in a pregnant woman. Eur J Obstet Gynecol Reprod Biol. 1996; 70:197-200.

46. Hameed AB, Tummala PP, Goodwin TM, et al. Unstable angina during pregnancy in two patients with premature coronary atherosclerosis and aortic stenosis in association with familial hypercholesterolemia. Am J Obstet Gynecol. 2000; 182:1152-1155.

47. Hands ME, Johnson MD, Saltzman DH, Rutherford JD. The cardiac, obstetric, and anesthetic management of pregnancy complicated by acute myocardial infarction. J Clin Anesth. 1990; 2:258-268.

48. Hankins GD, Wendel GD,Jr, Leveno KJ, Stoneham J. Myocardial infarction during pregnancy: A review. Obstet Gynecol. 1985; 65:139-146.

49. Hoppe UC, Beuckelmann DJ, Bohm M, Erdmann E. A young mother with severe chest pain. Heart. 1998; 79:205.

50. Houck PD, Strimel WJ, Gantt DS, Linz WJ. Should we establish a new protocol for the treatment of peripartum myocardial infarction? Tex Heart Inst J. 2012; 39:244-248.

51. Iaccarino D, Monopoli D, Rampino KC, Sangiorgi GM, Modena MG. Acute ST elevation myocardial infarction in early puerperium due to left main coronary thrombosis in a woman with thrombophilic state: A case report. J Cardiovasc Med (Hagerstown). 2010; 11:758-761.

52. Iadanza A, Del Pasqua A, Barbati R, et al. Acute ST elevation myocardial infarction in pregnancy due to coronary vasospasm: A case report and review of literature. Int J Cardiol. 2007; 115:81-85.

53. Janion M, Sielski J, Janion-Sadowska A. Myocardial infarction in pregnant women--case reports. Int J Cardiol. 2007; 121:207-209.

54. Jimenez Valero S, Garcia E, Delcan JL. Acute myocardial infarction during puerperium. report of two cases of multivessel involvement treated with primary coronary intervention. J Invasive Cardiol. 2005; 17:632-633.

55. Jungbluth A, Erbel R, Darius H, Rumpelt HJ, Meyer J. Paradoxical coronary embolism: Case report and review of the literature. Am Heart J. 1988; 116:879-885.

56. Kamran M, Suresh V, Ahluwalia A. Percutaneous transluminal coronary angioplasty (PTCA) combined with stenting for acute myocardial infarction in pregnancy. J Obstet Gynaecol. 2004; 24:701-702.

57. Kearney P, Singh H, Hutter J, Khan S, Lee G, Lucey J. Spontaneous coronary artery dissection: A report of three cases and review of the literature. Postgrad Med J. 1993; 69:940-945.

58. Klutstein MW, Tzivoni D, Bitran D, Mendzelevski B, Ilan M, Almagor Y. Treatment of spontaneous coronary artery dissection: Report of three cases. Cathet Cardiovasc Diagn. 1997; 40:372-376.

59. Knoess M, Otto M, Kracht T, Neis P. Two consecutive fatal cases of acute myocardial infarction caused by free floating thrombus in the ascending aorta and review of literature. Forensic Sci Int. 2007; 171:78-83.

60. Koul AK, Hollander G, Moskovits N, Frankel R, Herrera L, Shani J. Coronary artery dissection during pregnancy and the postpartum period: Two case reports and review of literature. Catheter Cardiovasc Interv. 2001; 52:88-94.

61. Kuczkowski KM. Cardiovascular complications of recreational cocaine use in pregnancy: Myth or reality? Acta Obstet Gynecol Scand. 2005; 84:100-101.

62. Kulka PJ, Scheu C, Tryba M, Oberheiden R, Zenz M. Coronary artery plaque disruption as cause of acute myocardial infarction during cesarean section with spinal anesthesia. J Clin Anesth. 2000; 12:335-338.

63. Kurum T, Soy M, Karahasanoglu E, Ozbay G, Sayin NC. A case of primary antiphospholipid syndrome who developed acute myocardial infarction followed by early-onset pre-eclampsia. Clin Rheumatol. 2003; 22:160-161.

64. Laudanski K, Euliano T. Peripartum acute coronary syndrome in an otherwise healthy patient. J Clin Anesth. 2011; 23:661-665.

65. Lerakis S, Manoukian S, Martin RP. Transesophageal echo detection of postpartum coronary artery dissection. J Am Soc Echocardiogr. 2001; 14:1132-1133.

66. Liu SS, Forrester RM, Murphy GS, Chen K, Glassenberg R. Anaesthetic management of a parturient with myocardial infarction related to cocaine use. Can J Anaesth. 1992; 39:858-861.

67. Livingston JC, Mabie BC, Ramanathan J. Crack cocaine, myocardial infarction, and troponin I levels at the time of cesarean delivery. Anesth Analg. 2000; 91:913-5, table of contents.

68. Mabie WC, Anderson GD, Addington MB, Reed CM,Jr, Peeden PZ, Sibai BM. The benefit of cesarean section in acute myocardial infarction complicated by premature labor. Obstet Gynecol. 1988; 71:503-506.

69. Madu EC, Kosinski DJ, Wilson WR, Burket MW, Fraker TD,Jr, Ansel GM. Two-vessel coronary artery dissection in the peripartum period. case report and literature review. Angiology. 1994; 45:809-816.

70. Maeder M, Ammann P, Drack G, Rickli H. Pregnancy-associated spontaneous coronary artery dissection: Impact of medical treatment. case report and systematic review. Z Kardiol. 2005; 94:829-835.

71. Majdan JF, Walinsky P, Cowchock SF, Wapner RJ, Plzak L,Jr. Coronary artery bypass surgery during pregnancy. Am J Cardiol. 1983; 52:1145-1146.

72. Mak KH, Chee JJ. Myocardial infarction and HELLP: A case of heightened vasomotor reactivity. Int J Cardiol. 2004; 97:151-152.

73. Makkonen M, Hietakorpi S, Orden MR, Saarikoski S. Myocardial infarction during pregnancy. Eur J Obstet Gynecol Reprod Biol. 1995; 58:81-83.

74. Marcoff L, Popescu A, Price L, et al. Spontaneous coronary artery dissection in a postpartum woman presenting with chest pain. Am J Emerg Med. 2010; 28:641.e5-641.e7.

75. Martins RP, Leurent G, Corbineau H, et al. Coronary angiography of pregnancy-associated coronary artery dissection: A high-risk procedure. Cardiovasc Revasc Med. 2010; 11:182-185.

76. McAdams SA, Maguire FE. Unusual manifestations of peripartal cardiac disease. Crit Care Med. 1986; 14:910-912.

77. McHugh MJ, Taubman MR. Postpartum myocardial infarction: A rehabilitation challenge. J Cardiovasc Nurs. 1990; 4:57-63.

78. McKechnie RS, Patel D, Eitzman DT, Rajagopalan S, Murthy TH. Spontaneous coronary artery dissection in a pregnant woman. Obstet Gynecol. 2001; 98:899-902.

79. McKeon VA, Perrin KO. The pregnant woman with a myocardial infarction: Nursing diagnosis. Dimens Crit Care Nurs. 1989; 8:92-100.

80. Moore AD, Hill J. Myocardial infarction and pregnancy: A case report. AANA J. 2012; 80:32-36.

81. Movsesian MA, Wray RB. Postpartum myocardial infarction. Br Heart J. 1989; 62:154-156.

82. Nabatian S, Quinn P, Brookfield L, Lakier J. Acute coronary syndrome and preeclampsia. Obstet Gynecol. 2005; 106:1204-1206.

83. Nallamothu BK, Saint M, Saint S, Mukherjee D. Clinical problem-solving. double jeopardy. N Engl J Med. 2005; 353:75-80.

84. Newell CP, Seller C, Vizhi M, Turner N. Case report: Spontaneous coronary artery dissection during elective caesarean section under spinal anaesthesia. Anaesthesia. 2011; 66:615-619.

85. O'Donnell M, Meecham J, Tosson SR, Ward S. Ventricular fibrillation and reinfarction in pregnancy. Postgrad Med J. 1987; 63:1095-1096.

86. Oki KN, Sinha R, Lee AW, Baker CJ. Emergency off-pump revascularization for left main dissection in pregnancy. Am Surg. 2011; 77:253-255.

87. Ottman EH, Gall SA. Myocardial infarction in the third trimester of pregnancy secondary to an aortic valve thrombus. Obstet Gynecol. 1993; 81:804-805.

88. Pauleta JR, Clode N, Tuna M, Graca LM. Acute myocardial infarction in pregnancy recurring in the puerperium. J Obstet Gynaecol. 2007; 27:520-521.

89. Phillips LM, Makaryus AN, Beldner S, Spatz A, Smith-Levitin M, Marchant D. Coronary artery dissection during pregnancy treated with medical therapy. Cardiol Rev. 2006; 14:155-157.

90. Pierre-Louis B, Singh P, Frishman WH. Acute inferior wall myocardial infarction and percutaneous coronary intervention of the right coronary during active labor: A clinical report and review of the literature. Cardiol Rev. 2008; 16:260-268.

91. Raber L, Meier B, Steiger VS, Gugger M, Vogel R. Peripartal myocardial infarction caused by placenta embolus. Circulation. 2011; 124:e26-7.

92. Rademacher W, Lauten A, Lauten A, Ragoschke-Schumm A, Figulla HR. Postpartum unmasking of a severe triple-vessel-disease with acute myocardial infarction. Clin Res Cardiol. 2010; 99:463-466.

93. Rahman S, Abdul-Waheed M, Helmy T, et al. Spontaneous left main coronary artery dissection complicated by pseudoaneurysm formation in pregnancy: Role of CT coronary angiography. J Cardiothorac Surg. 2009; 4:15.

94. Rajab TK, Khalpey Z, Kraemer B, Resnic FS, Gallegos RP. Recurrent post-partum coronary artery dissection. J Cardiothorac Surg. 2010; 5:78.

95. Ramineni R, Daniel GK. Association of a patent foramen ovale with myocardial infarction and pulmonary emboli in a peripartum woman. Am J Med Sci. 2010; 340:326-328.

96. Rensing BJ, Kofflard M, van den Brand MJ, Foley DP. Spontaneous dissections of all three coronary arteries in a 33-week-pregnant woman. Catheter Cardiovasc Interv. 1999; 48:207-210.

97. Rifai L, Trabolsi M, Dia M. Coronary embolus complicating peripartum cardiomyopathy. J Invasive Cardiol. 2011; 23:E237-40.

98. Sabatine MS, Jaffer FA, Staats PN, Stone JR. Case records of the massachusetts general hospital. case 28-2010. A 32-year-old woman, 3 weeks post partum, with substernal chest pain. N Engl J Med. 2010; 363:1164-1173.

99. Sage MD, Koelmeyer TD, Smeeton WM. Fatal postpartum coronary artery dissection. A light- and electron-microscope study. Am J Forensic Med Pathol. 1986; 7:107-111.

100. Salam AM. Acute myocardial infarction in the first trimester of pregnancy. Asian Cardiovasc Thorac Ann. 2005; 13:175-177.

101. Salem DN, Isner JM, Hopkins P, Konstam MA. Ergonovine provocation in post partum myocardial infarction. Angiology. 1984; 35:110-114.

102. Samuels LE, Kaufman MS, Morris RJ, Brockman SK. Postpartum coronary artery dissection: Emergency coronary artery bypass with ventricular assist device support. Coron Artery Dis. 1998; 9:457-460.

103. Saxena R, Nolan TE, von Dohlen T, Houghton JL. Postpartum myocardial infarction treated by balloon coronary angioplasty. Obstet Gynecol. 1992; 79:810-812.

104. Schiff JH, Gries A, Ehehalt R, Elsaesser M, Katus HA, Meyer FJ. A pregnant woman with acute myocardial infarction due to coronary artery dissection: Pre-hospital and in-hospital management. Resuscitation. 2007; 73:467-474.

105. Schumacher B, Belfort MA, Card RJ. Successful treatment of acute myocardial infarction during pregnancy with tissue plasminogen activator. Am J Obstet Gynecol. 1997; 176:716-719.

106. Sebastian C, Scherlag M, Kugelmass A, Schechter E. Primary stent implantation for acute myocardial infarction during pregnancy: Use of abciximab, ticlopidine, and aspirin. Cathet Cardiovasc Diagn. 1998; 45:275-279.

107. Shahabi S, Smith NA, Chanana C, Abbott JD, Copel J, Setaro JF. Pregnancy-associated myocardial infarction: A report of two cases and review of the literature. Ups J Med Sci. 2008; 113:325-330.

108. Sharma AM, Yactine H, Vedala G. Coronary vasospasm in a postpartum woman. J Invasive Cardiol. 2011; 23:E31-3.

109. Shaver PJ, Carrig TF, Baker WP. Postpartum coronary artery dissection. Br Heart J. 1978; 40:83-86.

110. Sherif HM, Nguyen HC, Sarter BH, et al. Spontaneous coronary dissection in late pregnancy: A multidisciplinary approach to management. Ann Thorac Surg. 2008; 85:1793-1794.

111. Silberman S, Fink D, Berko RS, Mendzelevski B, Bitran D. Coronary artery bypass surgery during pregnancy. Eur J Cardiothorac Surg. 1996; 10:925-926.

112. Skoura A, Michaelides M, Trikas A. Post-preeclampsia acute myocardial infarction during puerperium in a woman with normal coronary vessels. Hellenic J Cardiol. 2008; 49:55-58.

113. Spencer J, Gadalla F, Wagner W, Blake J. Caesarean section in a diabetic patient with a recent myocardial infarction. Can J Anaesth. 1994; 41:516-518.

114. Stefanovic V, Ulander VM, Graner M, Kupari M, Kaaja R. Myocardial infarction in early pregnancy. J Perinat Med. 2004; 32:541-542.

115. Tang AT, Cusimano RJ. Spontaneous coronary artery dissection complicating midterm pregnancy. Ann Thorac Surg. 2004; 78:e35.

116. Tatham K, Hughes-Roberts Y, Davies S, Johnson M, Ashpole K, Cox M. Peripartum cardiac chest pain and troponin rise. Int J Obstet Anesth. 2010; 19:453-455.

117. Taylor GW, Moliterno DJ, Hillis LD. Peripartum myocardial infarction. Am Heart J. 1993; 126:1462-1463.

118. Togni M, Amann FW, Follath F. Spontaneous multivessel coronary artery dissection in a pregnant woman treated successfully with stent implantation. Am J Med. 1999; 107:407-408.

119. Trouton TG, Sidhu H, Adgey AA. Myocardial infarction in pregnancy. Int J Cardiol. 1988; 18:35-39.

120. Ulm MR, Obwegeser R, Ploeckinger B, Nowotny C, Pidlich J, Sinzinger H. A case of myocardial infarction complicating pregnancy--a role for prostacyclin synthesis stimulating plasma factor and lipoprotein (a)? Thromb Res. 1996; 83:237-242.

121. van de Putte P, Martens PR. Atraumatic cardiac arrest in apparently healthy young women. Eur J Emerg Med. 1995; 2:179-183.

122. Varadarajan P, Isaeff D, Pai RG. Prosthetic valve thrombosis presenting as an acute embolic myocardial infarction in a pregnant patient: Issues on anticoagulation regimens and thrombolytic therapy. Echocardiography. 2006; 23:774-779.

123. Vogiatzis I, Hadjimiltiades S, Sachpekidis V, Parcharidis G. Spontaneous coronary artery dissection and acute myocardial infarction during pregnancy. Hellenic J Cardiol. 2010; 51:74-80.

124. von Steinburg SP, Klein E, Langwieser N, Kastrati A, Schneider KT, Zohlnhofer D. Coronary stenting after myocardial infarction during twin pregnancy--a case report. Hypertens Pregnancy. 2011; 30:485-489.

125. Webber MD, Halligan RE, Schumacher JA. Acute infarction, intracoronary thrombolysis, and primary PTCA in pregnancy. Cathet Cardiovasc Diagn. 1997; 42:38-43.

126. Wittry MD, Zimmerman TJ, Janosik DL, Williams GA. Postpartum myocardial infarction in a patient with intermittent ventricular preexcitation. Am Heart J. 1989; 117:191-194.

127. Yla-Outinen A, Lyrenas S, Lantz P, Langhoff-Roos J. Myocardial infarction in pregnancy. A case report and review of the literature. Ups J Med Sci. 1989; 94:287-290.

128. Zaidi AN, Raman SV, Cook SC. Acute myocardial infarction in early pregnancy: Definition of myocardium at risk with noncontrast T2-weighted cardiac magnetic resonance. Am J Obstet Gynecol. 2008; 198:e9-e12.
